# FOXA1 Gene Expression for Defining Molecular Subtypes of Muscle-Invasive Bladder Cancer after Radical Cystectomy

**DOI:** 10.3390/jcm9040994

**Published:** 2020-04-02

**Authors:** Danijel Sikic, Markus Eckstein, Ralph M. Wirtz, Jonas Jarczyk, Thomas S. Worst, Stefan Porubsky, Bastian Keck, Frank Kunath, Veronika Weyerer, Johannes Breyer, Wolfgang Otto, Sebastien Rinaldetti, Christian Bolenz, Arndt Hartmann, Bernd Wullich, Philipp Erben

**Affiliations:** 1 Department of Urology and Pediatric Urology, University Hospital Erlangen, Friedrich-Alexander University Erlangen-Nuremberg, 91054 Erlangen, Germany; bastian.keck@web.de (B.K.); frank.kunath@uk-erlangen.de (F.K.); bernd.wullich@uk-erlangen.de (B.W.); 2 Institute of Pathology, University Hospital Erlangen, Friedrich-Alexander University Erlangen-Nuremberg, 91054 Erlangen, Germany; markus.eckstein@uk-erlangen.de (M.E.); veronika.weyerer@uk-erlangen.de (V.W.); arndt.hartmann@uk-erlangen.de (A.H.); 3 STRATIFYER Molecular Pathology GmbH, 50935 Cologne, Germany; ralph.wirtz@STRATIFYER.de; 4 Department of Urology, University Medical Centre Mannheim, Medical Faculty Mannheim, University of Heidelberg, 68167 Mannheim, Germany; jonas.jarczyk@umm.de (J.J.); thomas.worst@umm.de (T.S.W.); philipp.erben@medma.uni-heidelberg.de (P.E.); 5 Institute of Pathology, University Medical Centre Mannheim, Medical Faculty Mannheim, University of Heidelberg, 68167 Mannheim, Germany; stefan.porubsky@umm.de; 6 Department of Urology, University of Regensburg, 93053 Regensburg, Germany; johannes.breyer@ukr.de (J.B.); wolfgang.otto@ukr.de (W.O.); 7 Department of Hematology and Oncology, University Medical Centre Mannheim, Medical Faculty Mannheim, University of Heidelberg, 68167 Mannheim, Germany; sebastien.rinaldetti@medma.uni-heidelberg.de; 8 Department of Urology and Pediatric Urology, University Hospital Ulm, 89081 Ulm, Germany; christian.bolenz@uniklinik-ulm.de

**Keywords:** FOXA1, GATA3, KRT20, molecular markers, mRNA, muscle-invasive bladder cancer, PCR, urothelial carcinoma

## Abstract

It remains unclear how to implement the recently revealed basal and luminal subtypes of muscle-invasive bladder cancer (MIBC) into daily clinical routine and whether molecular marker panels can be reduced. The mRNA expression of basal (KRT5) and luminal (FOXA1, GATA3, KRT20) markers was measured by reverse transcription quantitative real-time polymerase chain reaction (RT-qPCR) and correlated to clinicopathological features, recurrence-free survival (RFS), disease-free survival (DFS), and overall survival (OS) in 80 patients with MIBC who underwent radical cystectomy. Additionally, the correlation of single markers with the basal and non-basal subtypes defined by a 36-gene panel was examined and then validated in the TCGA (The Cancer Genome Atlas) cohort. High expression of FOXA1 (*p* = 0.0048) and KRT20 (*p* = 0.0317) was associated with reduced RFS. In the multivariable analysis, only FOXA1 remained an independent prognostic marker for DFS (*p* = 0.0333) and RFS (*p* = 0.0310). FOXA1 expression (AUC = 0.79; *p* = 0.0007) was closest to the combined marker expression (AUC = 0.79; *p* = 0.0015) in resembling the non-basal subtype defined by the 36-gene panel. FOXA1 in combination with KRT5 may be used to distinguish the basal and non-basal subtypes of MIBC.

## 1. Introduction

Urothelial carcinoma of the bladder (UCB) is the 10th most common cancer worldwide, with an estimated 549,000 new cases and 200,000 deaths per year [1]. While the majority of patients have non-muscle-invasive bladder cancer (NMIBC), approximately 25% of patients with UCB have muscle-invasive bladder cancer (MIBC) or metastases at the time of diagnosis [2]. While radical cystectomy and platin-based chemotherapy have remained the therapeutic standard for MIBC and metastatic disease in the last few decades, the treatment and follow-up of MIBC continues to be very challenging [3,4]. Radical cystectomy is associated with high rates of perioperative morbidity and mortality [5], and approximately 50% of patients experience distant disease recurrence after cystectomy, mostly within the first two years, although later recurrences have also been reported [6,7,8]. The median survival of patients treated with cisplatin-based chemotherapy for metastatic disease ranges between nine and 26 months [3]. The need for lifelong surveillance as well as high treatment costs result in UCB being the most expensive cancer per patient from diagnosis to death in the US [9,10,11]. Given the high costs and poor outcome of patients with UCB, there is a high demand for novel molecular markers to improve diagnostics and serve as targets for new therapies.

In recent years, several independent groups have demonstrated the existence of distinct molecular subtypes in UCB comparable to the molecular subtypes in breast cancer [12,13,14,15,16]. It was also shown that these molecular subtypes were associated with different outcomes and responses to chemotherapy [15,17]. While these findings bear great potential to improve the diagnostics and treatment of UCB in the future, there are still many uncertainties. Based on genetic expression patterns, most groups defined the basal and luminal subtypes of UCB by measuring the expression of hundreds of genes, which is not conveyable into daily clinical practice because of the high cost and effort [12,13,14,15,16]. The identification of relevant surrogate markers is necessary for the easy and feasible implementation of the molecular subtyping of UCB into daily clinical routine, as is the case in breast cancer [18].

Moreover, the exact number and definition of clinically relevant subtypes remain unclear. A recent consensus meeting agreed on the structure and features of a basal-squamous-like subtype, which is characterized by the high expression of the keratins KRT5/6 and KRT14 as well as the low expression of the transcription factors FOXA1 and GATA3 [19], which are suggested to drive luminal cell biology in bladder cancer [20]. However, to date, there has been no agreement on the definition of other non-basal subtypes or the markers necessary to define them.

Recently, using a 36-gene panel quantified by NanoString nCounter (NanoString Technologies Germany GmbH, Hamburg, Germany) in patients with MIBC treated with radical cystectomy, we were able to discriminate three prognostically distinct molecular subtypes (basal, luminal, and infiltrated) [21]. In an attempt to further reduce the required marker panel, we previously analyzed the prognostic relevance of the mRNA expression of KRT5 and KRT20 as surrogate markers for the basal and luminal subtypes of UCB, respectively [22,23]. However, it remains unclear if such a reduced marker panel adequately mirrors subtypes defined by larger marker panels. 

In the present study, we investigated the association of the mRNA expression of suggested surrogate markers for the basal (KRT5) and luminal (FOXA1, GATA3, KRT20, androgen receptor (AR)) subtypes of MIBC with clinical and pathological characteristics and survival. Furthermore, the association of the surrogate markers with the subtypes defined by the previously established 36-gene panel was examined with the intent to reduce marker panels for the non-basal subtypes [21]. 

## 2. Materials and Methods

### 2.1. Patient Population and Histological Assessment

In this study, we retrospectively analyzed tissue samples and clinical data from 80 patients with MIBC (stage pT2–pT4) who were treated between 1998 and 2006 with radical cystectomy and bilateral lymphadenectomy at the Department of Urology of the Medical Faculty Mannheim (Mannheim, Germany). Only patients who were treated with curative intention were included. All patients with metastases (*n* = 7) or unresectable (*n* = 1) tumors at the time of diagnosis were excluded, leaving a total of 73 patients to be included in this analysis. None of the patients received neoadjuvant or adjuvant therapy. The median follow-up time was 24 months (range: 1–184 months). All patients gave written informed consent. The study was approved by the relevant institutional review board at the Medical Faculty Mannheim under numbers 2013-517N-MA and 2016-814R-MA. Hematoxylin-eosin stained sections of the tumor samples were evaluated for pathological stage according to the 2010 TNM classification and were graded according to the common grading systems (WHO 1973, WHO 2016) by an experienced uropathologist (AH).

Expression data and clinicopathological information from the publicly available cancer genome atlas network (TCGA) cohort were used for validation (*n* = 406) [14]. Only patients with MIBC (T2–T4) were included, while all patients with no documented T or N stage and patients who received neoadjuvant therapy were excluded, leaving a total of 365 patients to be included in the analysis. Based on gene expression, the samples were clustered into five molecular subtypes (basal-squamous, luminal, luminal-papillary, luminal-infiltrated, and neuronal) [24].

### 2.2. Assessment of mRNA Expression by RT-qPCR

A reverse transcription quantitative real-time polymerase chain reaction (RT-qPCR)-based assessment was used for the objective quantification of FOXA1, GATA3, and AR mRNA expression, as previously described and performed with KRT5 and KRT20 [22,25]. In brief, RNA was extracted from a single 10-μm section of formalin-fixed paraffin embedded (FFPE) routine tissue using a commercially available bead-based extraction method (Xtract^®^ kit; STRATIFYER Molecular Pathology GmbH, Cologne, Germany). After a lysation and purification process, the nucleic acids were eluted and treated with DNase I. After the DNA was digested, the RNA eluates were stored at −80 °C until use.

One-step RT-qPCR was applied for the relative quantification of the mRNA expression of the genes of interest (FOXA1, GATA3 and AR) as well as the reference gene (Calmodulin 2 (CALM2)) by gene-specific TaqMan^®^-based assays using the SuperScript III PLATINUM One-Step, quantitative RT-PCR System (Invitrogen, Karlsruhe, Germany) on a Stratagene Mx3005p system (Agilent Technologies, Böblingen, Germany) with 30 minutes at 50 °C, two minutes at 95 °C, followed by 40 cycles of 15 seconds at 95 °C and 30 seconds at 60 °C as described previously [22,25].

Forty amplification cycles were applied, and the cycle threshold (Ct) values of the genes of interest and CALM2 for each sample were estimated as the mean value of the duplicate measurements. Ct values were then normalized against the mean expression levels of CALM2 using the 40-ΔCt method to ensure that normalized gene expression obtained by the test was proportional to the corresponding mRNA expression levels.

A set of 36 genes was previously quantified in 28 patients of this cohort using standard nCounter chemistry as previously described [21]. The nCounter assay was normalized using the geometric mean of six reference genes (CALM2, RPL37A, B2M, TUBB, GAPDH, and G6PD) and six internal positive controls, while negative background subtraction was conducted by eight negative internal controls, as previously described. Based on gene expression, urothelial carcinomas were assigned to one of three subtypes (basal, luminal, or infiltrated) [21]. Because of the small cohort size of 28 patients with available data on expression of the 36-gene panel, the subtypes were dichotomized into basal and non-basal subtypes.

The datasets for the TCGA cohort were downloaded as processed data from the open access cBioPortal database (http://www.cbioportal.org/study?id=blca_tcga#clinical) provided by the Memorial Sloan Kettering Cancer Center (New York, NY, USA). Gene expression analyses were based on paired-end RNA-Seq analysis on an Illumina HiSeq. All RSEM (RNA-Seq by Expectation Maximization) values were log2 transformed [24]. 

### 2.3. Statistical Methods

Correlation between variables was investigated by Spearman’s rank correlation coefficient, Wilcoxon/Kruskal–Wallis test, or Fisher’s exact test, whichever was appropriate. In addition, the cohort was stratified into patients with high or low marker expression using the median mRNA expression of KRT5, KRT20, FOXA1, GATA3, and AR as objective cut-offs. Recurrence-free survival (RFS), disease-free survival (DFS), and overall survival (OS) were analyzed by the Kaplan–Meier method and log-rank test. Univariable and multivariable analyses were performed by a Cox proportional hazards regression model. Receiver operating characteristic (ROC) curve analyses were used to measure the correlation between the markers and molecular subtypes. 

Statistical analysis was performed with JMP SAS 13.0 (SAS Institute, Cary, NC, USA) or Graph Pad Prism 5 (GraphPad Software Inc., La Jolla, CA, USA). All *p*-values were two-sided, and a *p*-value <0.05 was considered to be significant.

## 3. Results

### 3.1. Association of the Surrogate Markers with Clinicopathological Features

**Figure 1:**
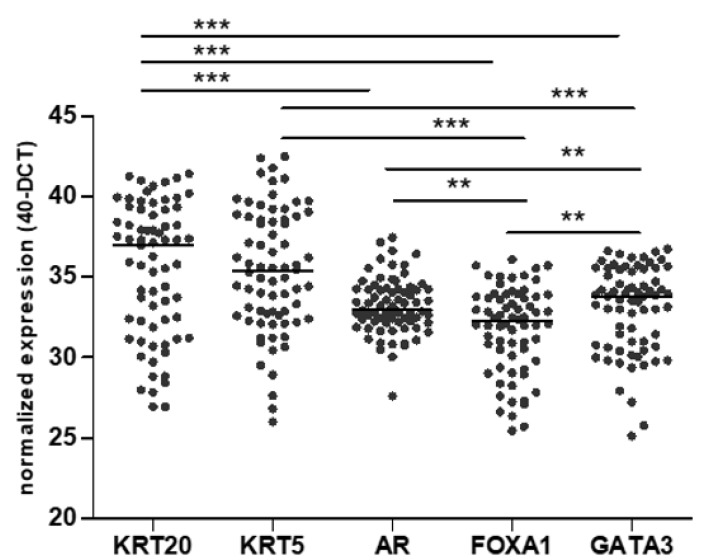
Distribution of normalized mRNA expression of KRT5, KRT20, FOXA1, GATA3, and androgen receptor (AR) in the Mannheim cohort (** *p* < 0.01; *** *p* < 0.001).

The characteristics of the included patients are summarized in Table 1. The median mRNA expression of all analyzed markers is shown in Figure 1.

**Figure 2:**
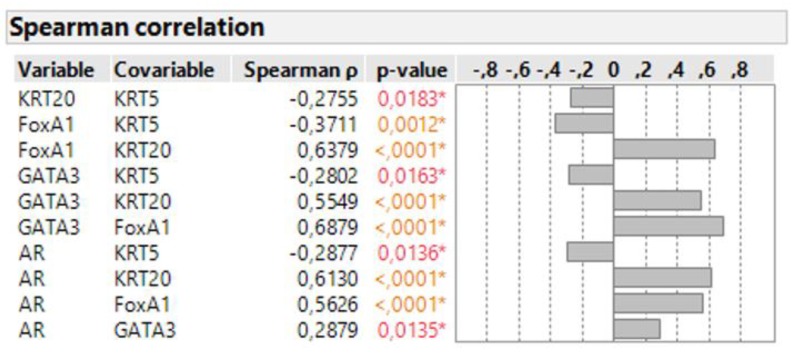
Spearman correlation of all measured markers (* *p* < 0.05).

Spearman correlation demonstrated a significant positive association of FOXA1 and GATA3 with the luminal marker KRT20 (Figure 2). All three luminal markers showed a significantly negative association with the basal marker KRT5. Moreover, AR also showed a significantly positive association with all luminal markers, while the association between AR and KRT5 was negative. 

The Wilcoxon/Kruskal–Wallis test showed significant positive associations between grade and both KRT5 (0.0388) and GATA3 (*p* = 0.0133). There were no significant associations with tumor stage, nodal status, sex, or age.

### 3.2. Association of the Surrogate Markers with Survival

**Figure 3:**
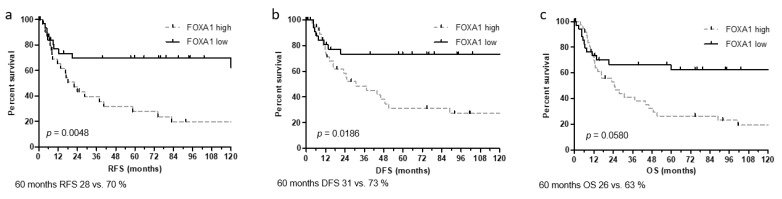
Kaplan Meier analysis for recurrence-free survival (RFS) (**a**), disease-free survival (DFS) (**b**) and overall survival (OS) (**c**) based on FOXA1 mRNA expression within the Mannheim cohort.

For survival analysis, the median mRNA expression levels of each marker were used as an objective cut-off to stratify patients with high and low marker expression. Kaplan–Meier analysis indicated that high KRT20 (*p* = 0.0317) and FOXA1 (*p* = 0.0048) expression was associated with significantly reduced RFS. GATA3 (*p* = 0.0629) and KRT5 (*p* = 0.0513) were not significantly associated with RFS. When analyzing the association with DFS and OS, only FOXA1 was significantly associated with reduced DFS (*p* = 0.0186) (Figure 3), while KRT5, KRT20, and GATA3 showed no associations with OS or DFS. AR mRNA expression showed no relevant associations with RFS, DFS, or OS.

In univariable Cox regression analysis, positive nodal status, tumor stage, and expression of FOXA1 and KRT20 were associated with worse outcome (Table 2, Table 3 and Table 4). In the multivariable analysis, of all examined markers, only FOXA1 remained an independent prognostic marker for DFS (*p* = 0.0333) and RFS (*p* = 0.0310) (Table 2, Table 3 and Table 4). When analyzing patients with pure urothelial carcinomas (*n* = 43) and patients with histologic variants (*n* = 30) separately, we found an improved survival for patients with low FOXA1 expression and pure urothelial carcinomas but not histologic variants, which might be attributed to the low number of 30 patients and high heterogeneity of the histologic variants (Figure S1). 

There was no association between FOXA1 expression and DFS in the TCGA cohort (Figure S2).

### 3.3. Correlation of Surrogate Markers with Molecular Subtypes Defined by Multigene Panels

**Figure 4:**
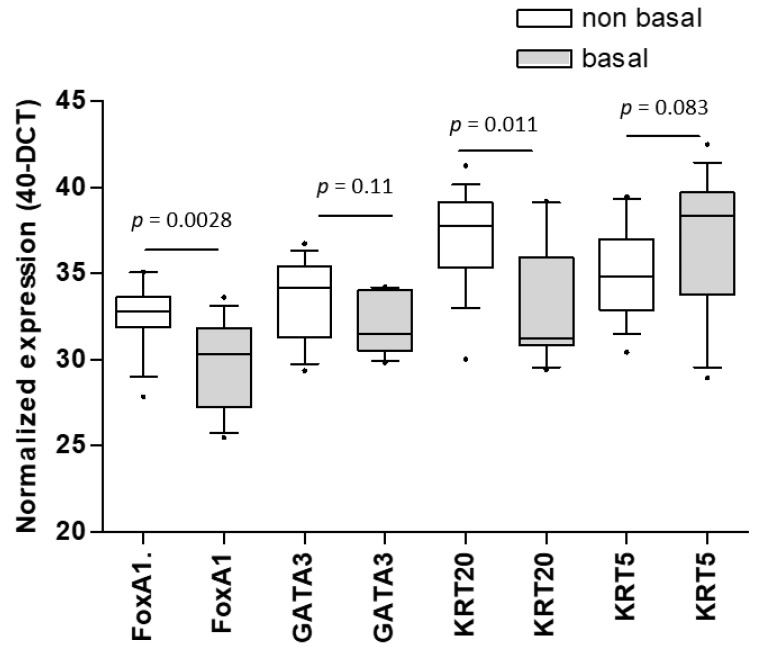
Distribution of FOXA1, GATA3, KRT20, and KRT5 within the basal and non-basal subtypes defined by a 36-gene panel within the Mannheim cohort showing significantly higher expression of FOXA1 and KRT20 in the non-basal subtype.

In the Mannheim cohort, a total of 28 patients were clustered into three molecular subtypes (basal, luminal, and infiltrated) according to the expression of a 36-gene panel previously quantified with nCounter [21]. Because of the small cohort with available data on molecular subtypes, we dichotomized the subtypes into basal and non-basal subtypes in the present study. The Mann–Whitney test showed that FOXA1 (*p* = 0.0028) and KRT20 (*p* = 0.011) expression was significantly higher in the non-basal subtype (Figure 4). The expression of KRT5 (*p* = 0.083) and GATA3 (*p* = 0.11) was not significantly different between the two subtypes in the Mannheim cohort. In the TCGA cohort, all three luminal markers had significantly higher expression in the non-basal subtype, while KRT5 was significantly higher in the basal subtype (each *p* < 0.0001).

Using the median marker expression as the cut-off, ROC analyses showed a high but not significant correlation of KRT5 with the basal subtype (AUC = 0.65; *p* = 0.097) in the Mannheim cohort. FOXA1 (AUC = 0.79; *p* = 0.0007) and KRT20 (AUC = 0.75; *p* = 0.0066) correlated significantly with the non-basal subtype, unlike GATA3 (AUC = 0.65; *p* = 0.097). The use of all three luminal markers combined showed no relevantly improved approximation to the non-basal subtype (AUC = 0.79; *p* = 0.0015) over the use of KRT20 or especially FOXA1 alone. Validation in the TCGA cohort showed that the use of FOXA1 alone (AUC = 0.77; *p* < 0.0001) achieved a high approximation to the non-basal subtype, similar to the use of all three markers combined (AUC = 0.79; *p* < 0.0001), while KRT5 achieved a close approximation to the basal subtype (AUC = 0.75; *p* < 0.0001). To exclude the possibility that the high association of KRT5 and FOXA1 with the basal and non-basal subtypes is mainly based on the central role of KRT5 and FOXA1 in the classification of subtypes using the 36-gene panel and the TCGA classification, we applied the BASE47 signature, which does not include KRT5 and FOXA1 for defining subtypes, on the TCGA cohort for validation [16]. This way, KRT5 and FOXA1 still showed a significantly higher distribution in the basal and luminal subtype, respectively (Figure S3). Furthermore, there was still a high association between the basal subtype and KRT5 (AUC = 0.72; *p* < 0.0001) and the luminal subtype and FOXA1 (AUC = 0.77; *p* < 0.0001). 

## 4. Discussion

With the advent of molecular subtyping in UCB, researchers and clinicians are faced with several problems, as was the case with molecular subtyping in breast cancer 15 years ago. First, apart from a basal squamous-like subtype, no consensus on the number and essential characteristics of other molecular subtypes has yet been reached [19]. As most groups have defined various numbers of luminal-like subtypes, these differences in labeling, subclassification and marker expression have hindered the general acceptance of these non-basal subtypes so far [12,14,16,21].

Second, it is necessary that subtypes are either prognostically or therapeutically relevant; otherwise, they are useless for daily clinical routine. For instance, analogous to breast cancer, a claudin-low subset of basal UCB was previously defined which demonstrated a similar outcome as regular basal UCB [16], therefore being of no interest for clinical routine.

Third, the analysis of hundreds of genes per patient currently used by most groups for the definition of their subtypes is too time-consuming and cost-intensive for easy transfer into a routine clinical setting, which is why a small set of surrogate markers per subtype has to be determined. In breast cancer, it was shown that the analysis of only four markers (estrogen receptor, progesterone receptor, HER2, and Ki-67) is enough to make a valid therapeutically relevant molecular classification [18,26]. In addition, a recent study in prostate cancer showed that the status of the PTEN gene alone matched a multigene panel to predict the risk of metastasis in patients treated with radical prostatectomy, allowing for more cost-saving diagnostics [27].

By analogy to previous findings in breast and prostate cancer, the goal of the present study was to identify surrogate markers for molecular subtypes with regard to their prognostic relevance and concordance with subtypes defined by multigene panels. Given that there is consensus about the basal subtype, we focused on non-basal subtypes. Therefore, we decided to analyze the mRNA expression of the two prominent luminal markers, FOXA1 and GATA3, together with the previously measured luminal marker KRT20 and the basal marker KRT5 [22], alongside AR as a potential target associated with the luminal subtype of MIBC [28].

Regarding the expression pattern, our results are in concordance with previous findings, as FOXA1 and GATA3 are strongly associated with KRT20 expression and show a negative association with KRT5 [12,16]. As before, we found AR to be associated with the luminal subtype in MIBC [28].

When comparing single-marker analysis to the subtypes defined by the 36-gene panel, FOXA1 and KRT20 expression was significantly higher in the non-basal subtype than in the basal subtype. Moreover, the analysis of only FOXA1 or KRT20 showed a similar high correlation with the 36-gene panel when compared to the analysis of FOXA1, GATA3 and KRT20 combined. The use of FOXA1 alone showed an almost identical AUC in the Mannheim (0.79) and TCGA (0.77) cohorts when compared to the use of all three luminal markers together (Mannheim 0.79; TCGA 0.76). FOXA1 is known to play a central role in urothelial differentiation. In addition, the low expression or loss of FOXA1 in basal tumors was described in the development of squamous cell carcinoma in preclinical models of bladder cancer, which is in concordance with the subtype association in the current study [29]. The nonsignificant correlation of GATA3 with the non-basal subtype and KRT5 with the basal subtype in the Mannheim cohort might be attributed to the small sample size of only 28 patients for whom the 36-gene panel results were available. The correlation of all markers with their respective subtype was confirmed in the TCGA cohort. The current results indicate that the measurement of one of the luminal markers, could be enough to determine a non-basal subtype, potentially allowing for more cost-saving diagnostics in daily clinical routine. 

When analyzing the prognostic relevance, high KRT5 showed a non-significant trend for prolonged RFS, while FOXA1 was the only luminal marker that remained an independent prognostic marker for reduced RFS when all markers were accounted for, which suggests that a combined analysis of all three luminal markers does not provide any additional prognostically relevant information. These results are in contrast to several previous studies that found the basal subtype to be associated with worse outcome [15,17]. With regard to FOXA1 in particular, Yuk and colleagues reported higher FOXA1 expression to be associated with a positive prognostic outcome using immunohistochemistry on tissue microarray slides [30]. One possible reason for this discrepancy might be the high percentage (41%) of histologic variants within the analyzed Mannheim cohort, which are often associated with poorer prognosis than pure urothelial carcinomas and demonstrate a higher expression of luminal markers such as FOXA1 [20,31]. Furthermore, some studies show an association between the high expression of luminal markers such as KRT20 and high tumor stage, grade and micrometastasis, which are known to be associated with worse survival, which further indicates a luminal patient group with impaired survival [32,33]. On the other hand, no prognostic relevance for any of the luminal markers could be shown in the TCGA cohort. Tumor heterogeneity, which was not accounted for in the current study, might also be a factor for these contradictory results [34]. Further studies are necessary to clarify the prognostic role of molecular markers in MIBC.

Moreover, while the analysis of only one basal and one luminal marker seems to be enough to make a valid distinction between the basal and non-basal subtypes, additional markers still might be necessary for subclassification. In a comprehensive molecular analysis of MIBC within the TCGA cohort, Robertson et al. were able to identify three distinct luminal subclasses, with the luminal and luminal-infiltrated subtypes being associated with reduced survival compared to the luminal-papillary subtype [24]. All three luminal subtypes showed comparable expression patterns of FOXA1, GATA3, and KRT20 but differed with regard to FGFR3 mutations, lymphocytic infiltration, smooth muscle gene signatures, and uroplakin expression. Differences in luminal subclasses were not accounted for in our current study. The analysis of one or two additional markers (for instance, the epithelial–mesenchymal transition (EMT) markers TWIST1 or SNAI1) might be enough to draw clinically relevant conclusions. Moreover, a rare neuronal/neuroendocrine-like subtype associated with poor survival has previously been described in approximately 5% of patients with MIBC [24,35]. These tumors are mainly characterized by the upregulation of genes of neuroendocrine origin, such as TUBB2B but can also express FOXA1 and GATA3. Markers to distinguish these neuronal/neuroendocrine-like subtypes from luminal subtypes still have to be defined.

As previously mentioned, with a total of 73 included patients, our cohort is relatively small compared to other multicentric studies. On the other hand, given that this is a single center study, we have exact information on treatment modalities, which is necessary to interpret the prognostic relevance of markers, although some data on salvage therapies is missing due to the retrospective nature of the study. However, this is also the case in the TCGA cohort, where no precise information on the treatment modality is provided, rendering statements about prognosis even more difficult. 

## 5. Conclusions

In conclusion, we were able to demonstrate that the measurement of only one of the prominent luminal markers alongside KRT5 as the basal marker is enough to make a valid distinction between the basal and non-basal subtypes and potentially draw prognostically relevant conclusions. Given the closest concordance with subtypes defined by multigene panels as well as strongest prognostic relevance, FOXA1 seems to be the marker best suited as a surrogate marker to distinguish the non-basal subtypes from the basal subtypes. The measurement of FOXA1, GATA3, and KRT20 combined does not provide any additional relevant information. However, additional studies are necessary to further clarify the prognostic role of molecular markers in MIBC. Moreover, surrogate markers for the further subclassification of the luminal subtype still have to be defined.

## Figures and Tables

**Table 1:** Patient characteristics of the Mannheim cohort.

Patient Characteristic	*n* (%)
Tumor stage	
T2	18 (24.7)
T3	43 (58.9)
T4	12 (16.4)
Nodal status	
N0	44 (60.3)
N positive	29 (39.7)
Sex	
male	54 (74.0)
female	19 (26.0)
Grade (WHO 1973)	
G2	15 (20.5)
G3	58 (79.5)
Age	
<70	47 (64.4)
≥70	26 (35.6)
Histology	
Pure urothelial carcinoma	43 (58.9)
Histologic variants	30 (41.1)
Squamous cell	10 (13.7)
Sarcomatoid	7 (9.6)
Micropapillary	5 (6.9)
Small cell	3 (4.1)
Adenocarcinoma	2 (2.7)
Neuroendocrine	1 (1.4)
Nested	1 (1.4)
Plasmacytoid	1 (1.4)

**Table 2:** Univariable and multivariable Cox regression analyses for RFS (Recurrence-free survival), accounting for all five analyzed markers and clinicopathological features.

Parameter	Univariable		Multivariable	
	Hazard Ratio	*p*-Value	Hazard Ratio	*p*-Value
Tumor stage				
T3/4 vs.	1.6203	0.2156		
T2				
Nodal status				
N+ vs.	2.8737	**0.0027**	2.4330	**0.0146**
N0				
Sex				
male vs.	1.0155	0.9698		
female				
Grade (WHO 1973)				
G2 vs.	0.4709	0.8186		
G3				
Age				
<70 vs.	0.8428	0.6454		
≥70				
KRT5				
>median vs.	0.5081	0.0513		
<median				
KRT20				
>median vs.	2.1233	**0.0317**	1.2927	0.5083
<median				
FOXA1				
>median vs.	2.7209	**0.0048**	2.2670	**0.0310**
<median				
GATA3				
>median vs.	1.9211	0.0629		
<median				
AR				
>median vs.	1.6121	0.1670		
<median				

Multivariable analysis was performed only for significant parameters in univariable analysis (significant values in bold).

**Table 3:** Univariable and multivariable Cox regression analyses for DFS (Disease-free survival), accounting for all five analyzed markers and clinicopathological features.

Parameter	Univariable		Multivariable	
	Hazard Ratio	*p*-Value	Hazard Ratio	*p*-Value
Tumor stage				
T3/4 vs.	2.0330	0.0926		
T2				
Nodal status				
N+ vs.	2.7727	**0.0044**	2.6057	**0.0077**
N0				
Sex				
male vs.	1.5435	0.2715		
female				
Grade (WHO 1973)				
G2	1.1484	0.7558		
G3				
Age				
<70	0.8833	0.7404		
≥70				
KRT5				
>median vs.	0.6999	0.3072		
<median				
KRT20				
>median vs.	1.3249	0.4235		
<median				
FOXA1				
>median vs.	2.3617	**0.0186**	2.1946	**0.0333**
<median				
GATA3				
>median vs.	1.2429	0.5359		
<median				
AR				
>median vs.	1.3717	0.3670		
<median				

Multivariable analysis was performed only for significant parameters in univariable analysis (significant values in bold).

**Table 4:** Univariable and multivariable Cox regression analyses for OS (Overall survival), accounting for all five analyzed markers and clinicopathological features.

Parameter	Univariable		Multivariable	
	Hazard Ratio	*p*-Value	Hazard Ratio	*p*-Value
Tumor stage				
T3/4 vs.	2.0601	**0.0048**	1.7819	0.1251
T2				
Nodal status				
N+ vs.	2.2720	**0.0086**	2.0806	**0.0206**
N0				
Sex				
male vs.	1.3586	0.3820		
female				
Grade (WHO 1973)				
G2 vs.	0.7678	0.4590		
G3				
Age				
<70 vs.	0.8253	0.5544		
≥70				
KRT5				
>median vs.	0.7012	0.2408		
<median				
KRT20				
>median vs.	1.1560	0.6334		
<median				
FOXA1				
>median vs.	1.7986	0.0580		
<median				
GATA3				
>median vs.	1.0844	0.7800		
<median				
AR				
>median vs.	1.3104	0.3728		
<median				

Multivariable analysis was performed only for significant parameters in univariable analysis (significant values in bold).

## Supplementary Materials

The following are available online at https://www.mdpi.com/2077-0383/9/4/994/s1, Figure S1: Kaplan–Meier analysis for RFS, DFS, and OS based on FOXA1 mRNA expression within the Mannheim cohort in patients with pure urothelial carcinoma and patients with histologic variants. Figure S2: Kaplan–Meier analysis for disease specific survival based on FOXA1 mRNA expression within the TCGA cohort. Figure S3: Distribution of normalized mRNA expression of FOXA1 within the basal and luminal subtypes defined by a 47-gene panel (BASE47) within the TCGA cohort.
