# Supplementary material for: FOXA1 Gene Expression for Defining Molecular Subtypes of Muscle-Invasive Bladder Cancer after Radical Cystectomy
Source: J Clin Med. 2020 Apr 2;9(4):994. doi: 10.3390/jcm9040994 (PMC7230662; doi:10.3390/jcm9040994)
Supplement: Supplementary file 1 [file jcm-09-00994-s001.pdf]

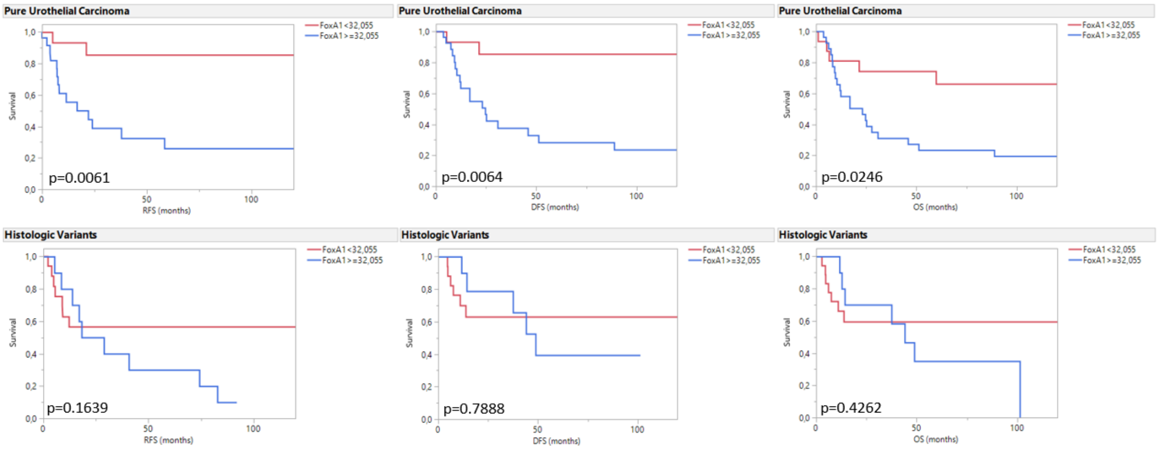

Figure S1. Kaplan–Meier analysis for RFS, DFS, and OS based on FOXA1 mRNA expression within the Mannheim cohort in patients with pure urothelial carcinoma and patients with histologic variants.

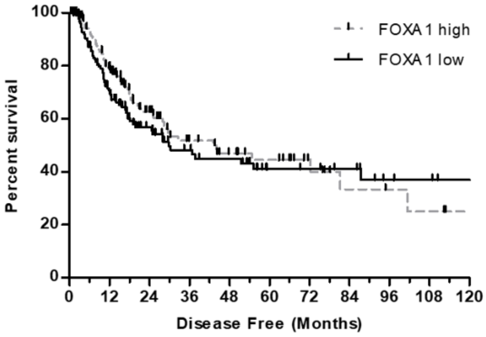

Figure S2. Kaplan–Meier analysis for disease specific survival based on FOXA1 mRNA expression within the TCGA cohort.

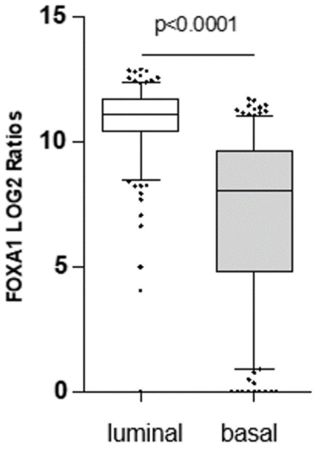

Figure S3. Distribution of normalized mRNA expression of FOXA1 within the basal and luminal subtypes defined by a 47-gene panel (BASE47) within the TCGA cohort.
